# Risk Factors for Postoperative Pulmonary Complications in Patients Undergoing Thoracotomy for Indications Other than Primary Lung Cancer Resection: A Multicenter Retrospective Cohort Study from the German Thorax Registry

**DOI:** 10.3390/jcm14051565

**Published:** 2025-02-26

**Authors:** Wolfgang Baar, Axel Semmelmann, Florian Anselm, Torsten Loop, Sebastian Heinrich, for the Working Group of the German Thorax Registry

**Affiliations:** 1 Department of Anesthesiology and Critical Care, Medical Center-University of Freiburg, Faculty of Medicine, University of Freiburg, 79106 Freiburg, Germany; axel.semmelmann@uniklinik-freiburg.de (A.S.); florian.anselm@uniklinik-freiburg.de (F.A.); torsten.loop@uniklinik-freiburg.de (T.L.); sebastian.heinrich@uniklinik-freiburg.de (S.H.); 2 German Society of Anesthesiology and Intensive Care Medicine, 10115 Nürnberg, Germany; 3 German Society for Thoracic Surgery e.V., 10117 Berlin, Germany

**Keywords:** postoperative pulmonary complications, thoracotomy, indications for thoracotomy other than lung cancer, in-hospital mortality of thoracotomy

## Abstract

**Background:** Postoperative pulmonary complications (PPCs) are the most common complications following lung surgery and can lead to increased postoperative mortality. In this study, we examined the incidence of PPCs, the in-hospital mortality rate, and the risk factors associated with PPCs in patients undergoing open thoracotomy lung resection (OTLR) for reasons other than primary lung cancer. **Methods**: Data from this multicenter, retrospective study involving 1.368 patients were extracted from the German Thorax Registry and analyzed using univariate and multivariable statistical methods. **Results**: In total, 278 patients showed at least one PPC. The presence of PPCs was associated with a significantly higher in-hospital mortality rate (7.2% vs. 1.5%; *p* = 0.000). Multivariable stepwise logistic regression analysis showed absolute age (OR 1.02) and BMI ≤ 19 (OR 2.6) as independent patient-specific risk factors. Significant preoperative risk factors included re-thoracotomy (OR 4.0) and FEV_1_ < 60% (OR 2.5). Procedure-related independent risk factors for PPCs included a surgical duration surpassing 195 min (OR 2.7), the continuation of invasive ventilation post-surgery (OR 3.8), and an intraoperative infusion of crystalloids greater than 6 mL/kg/h (OR 1.8). **Conclusions**: Optimizing intraoperative fluid therapy and on-table extubation when possible may reduce the incidence of PPCs and associated mortality.

## 1. Introduction

Beyond lung resection for primary bronchial carcinoma, thoracic surgeries are also performed for the resection of lung metastases originating from various types of carcinomas [1]. Advances in surgical techniques and the development of more effective chemotherapy regimens that expanded the role of surgery have gradually established lung metastasectomy as a standard therapeutic procedure for selected patients [2]. Oncologic and functional operability are the main criteria [2]. Pulmonary metastasectomy (PM) is an established treatment for various types of cancer, including colorectal cancer and renal cell carcinoma [3]. Postoperative pulmonary complications (PPCs) following lung surgery—no matter what indication—are the most frequent complication, with an incidence of up to 24% and causing up to 68% of unplanned ICU admissions [4,5,6]. Patients undergoing lung surgery are frequently older and have multiple comorbidities, leading to various complications and unplanned admission to the ICU, with consecutive increased mortality and length of hospital stay [4]. Most studies investigate postoperative pulmonary complications in patients receiving lung resection for primary lung cancer or patients with pleural empyema [7,8].

When respiratory failure following lung surgery requires noninvasive ventilation or endotracheal re-intubation with extended mechanical ventilation, both short-term and long-term mortality rates are elevated [9]. The underlying risk factors that contribute to an increased rate of PPCs have been investigated in previous studies. Commonly recognized factors include advanced age, a body mass index (BMI) of 30 kg/m^2^ or higher, pre-existing obstructive lung disease, and fluid overload. These factors have been shown to significantly impact the likelihood of experiencing PPCs following thoracic surgery [8,10,11,12,13].

In a multicenter, retrospective register study focused on identifying various risk factors for PPCs following VATS lung resection, we discovered that prolonged surgical duration and the administration of crystalloids exceeding 6 mL/kg/h during the surgery were significant modifiable risk factors contributing to the occurrence of PPCs [14]. This study focused exclusively on VATS lung resection. To further investigate, we conducted a multicenter retrospective analysis to identify risk factors for PPCs in patients undergoing open thoracotomy lung resection (OTLR) due to lung cancer. Our findings revealed a statistically significant and clinically important increase in mortality for patients who experienced two or more PPCs compared to those who had one or no PPC. We could demonstrate that performing extubation on the table, if feasible, using continuous neuroaxial analgesia, timing surgeries when patients are infection-free, and limiting crystalloid fluid intake to no more than 6 mL/kg/h are independent, modifiable risk factors [8].

The existing literature focuses most on PPCs in lung surgery because of bronchial carcinoma or, on rare occasions, on metastasectomy patients—all the other indications for lung surgery. Therefore, PPCs are underrepresented.

The underlying aim of the current study was to detect risk factors for PPCs in patients suffering OTLR for reasons other than primary lung cancer resection and to determine whether anesthesia-related factors such as regional anesthesia or intraoperative fluid management significantly affect the incidence of PPCs.

## 2. Materials and Methods

### 2.1. Data Source

A multicenter retrospective cohort registry and database of patients who underwent non-cardiac thoracic surgery involving open thoracotomy between 2016 and 2020 was the data source. Data were identified and extracted from the 2021 database of the German Thorax Registry. This resulted in a total of 6.917 patients from four contributing centers who received non-cardiac thoracic surgery. The German Thorax Registry is an interdisciplinary and multicenter database initiated by the German Society of Anaesthesiology and Intensive Care Medicine (DGAI) and the German Society of Thoracic Surgery (DGT) [15]. The clinics involved send the collected data to a central database using a web-based application. The sent data are pseudonymized. That means that a sequence of numbers replaces the patient’s name. Completing the register datasets required a mandatory core dataset that included epidemiological data, surgical and anesthesiological parameters, and outcome-related parameters. Without the completeness of the core dataset, case entry could not be completed. In order to avoid selection bias due to missing data, the cases entered in the register were compared with the total number of treated cases per center. Participation in the registry was only possible if the participating centers provided data from the mandatory core dataset. This study adheres to the publication guidelines set by the German Thorax Registry, and our request for data analysis was formally approved and registered by its advisory board. The study was planned and designed in accordance with the initiative to enhance the reporting of observational studies in epidemiology (STROBE), utilizing the recommended checklist for epidemiological cohort studies (Supplementary Table S1) [16].

### 2.2. Ethics

Ethical approval for this study was granted by the Ethical Committee of the University Witten/Herdecke (Approval No.: 64/2014) on 24 June 2014. The approval, which was overseen by Chairperson Prof. P. W. Gaidzik, also extends to data collected beyond the year of approval.

### 2.3. Patients

**Figure 1:**
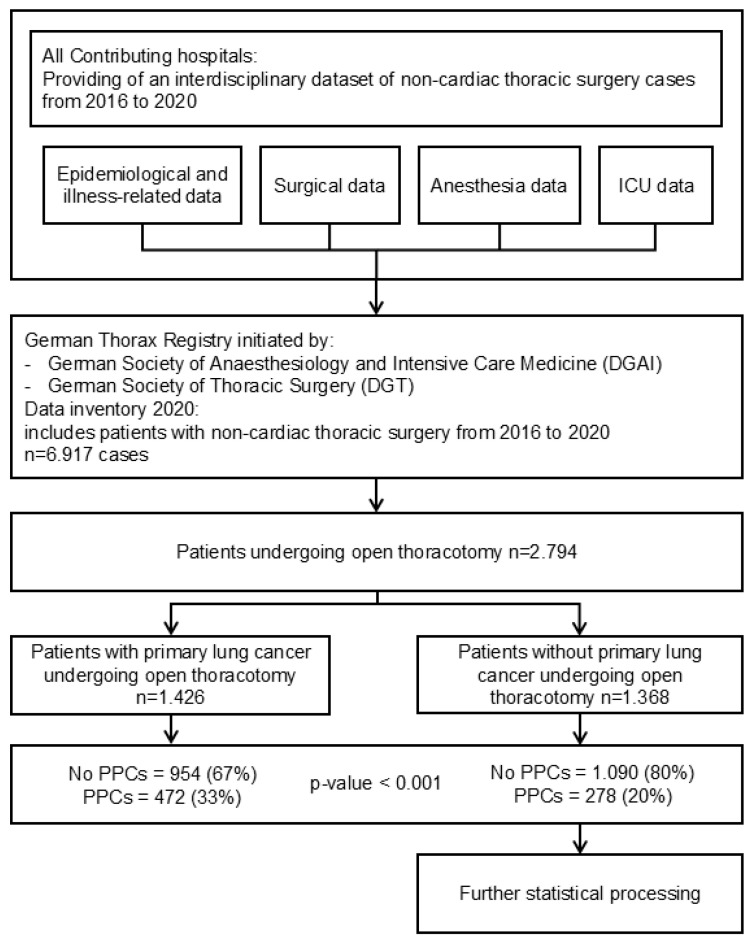
Selection of cases, design of studies, and incidence of PPC related to entities are detailed. Data are shown as patient counts (percentage). PPCs refers to postoperative pulmonary complications, ICU stands for intensive care unit, and DGAI is the German Society of Anaesthesiology and Intensive Care Medicine.

The study’s selection of cases is illustrated in Figure 1. Only patients who underwent thoracotomy for non-cardiac surgery were included. A total of 2.794 patients were selected for the study. They were then divided into two groups: one group consisted of 1.426 patients who underwent open thoracotomy and lung surgery for primary lung cancer, while the other group included 1.368 patients who received surgery for reasons other than primary lung cancer. Most patients of this last group received thoracotomy for resections of lung metastasis of other cancer entities (553, 40%) and benign tumors (393, 29%). Other indications were resections of other tumors (190, 14%), thoracotomies because of a septic focus (103, 8%), resection of mediastinal tumors (101, 7%), or surgical revisions (28, 2%). All patients received at least general anesthesia. During the open thoracotomy, one-lung ventilation (OLV) was implemented. The primary focus of this study was on PPCs. PPCs were defined as a composite outcome, including prolonged air leakage lasting seven days or more after surgery, postoperative pneumonia, the need for non-invasive ventilation (NIV) because of respiratory failure, the requirement for new chest drains after surgery, re-intubation as a result of respiratory failure or continued ventilation following surgery, pleural empyema, or the need for extracorporeal membrane oxygenation (ECMO). In our study, pneumonia was diagnosed according to the European perioperative clinical outcome (EPCO) definition. This definition includes criteria such as new pulmonary infiltrate, associated leukocytosis, fever, new purulent sputum, the need for antibiotic therapy, and increased oxygen demand via a face mask [17].

Figure 1 illustrates that patients who had lung resection for primary lung cancer experienced notably higher PPCs than those who did not. These results have already been published [8]. Therefore, we focused on patients who received open thoracotomy for tumor resection apart from primary lung cancer.

### 2.4. Parameters

The following list outlines the patient-specific and preoperative characteristics that were included in the univariate analysis: gender, body mass index (BMI), age, ASA score, smoking status, history of pulmonary infection within four weeks prior to surgery, FEV_1_ ≤ 60%, previous lung surgery, preoperative radio- and/or chemotherapy (RCT), TNM staging, preoperative hemoglobin level, preoperative C-reactive protein level > 3 mg/dL, and data from preoperative blood gas analysis.

Procedural characteristics included parameters related to surgery and anesthesia. Surgery-related characteristics encompassed a duration of at least 195 min, the volume of lung tissue resected, and the total intraoperative blood loss.

The anesthetic management was reviewed, detailing the specific anesthesia techniques used, including single-shot intercostal block (ICB), single-shot paravertebral block (PVB), continuous neuraxial techniques such as paravertebral catheter (PVC), and thoracic epidural anesthesia via a catheter (TEA). Additionally, the maintenance of anesthesia was achieved either through inhaled anesthetics or total intravenous anesthesia. The analysis also covered fluid and vasopressor therapy, which included an intraoperative crystalloid infusion rate of ≥6 mL/kg/h. It specified the total amounts of intraoperative crystalloids, colloids, packed red blood cells (PRBCs), and fresh frozen plasma used, as well as the requirement for intraoperative vasopressor therapy. Additionally, the ventilator settings were assessed during one-lung ventilation, focusing on the average positive end-expiratory pressure (PEEP) and the lowest inspiratory fraction of oxygen (FiO_2_). The amount of fluid infusion was according to the institutional standard operating procedures and the discretion of the attending anesthesiologist. All centers are focusing on protective ventilation. Further analysis addressed the necessity for partial ligation of the pulmonary artery and the requirement for ongoing invasive ventilation immediately after surgery.

### 2.5. Statistics

Univariate statistical analysis was conducted by dividing the cohort into two groups: patients with postoperative complications (PPCs) and patients without PPCs. Continuous variables were analyzed using the Mann–Whitney U test, while categorical variables were assessed using the chi-square (χ^2^) test. For the multivariable stepwise logistic regression analysis, only parameters that had a *p*-value < 0.05 in the univariate analysis were included to evaluate their impact on the incidence of PPCs. The volume of parenchyma that was resected was not included in the multivariable regression analysis because the oncological requirements determined it and it could not be influenced by patients, surgeons, or anesthesiologists. For the multivariable regression analysis, a stepwise forward approach was used, including the following parameters: ASA status ≥ 3, age, BMI ≤ 19, smoking cessation for at least 3 months, CRP levels < 3 mg/dL, history of re-thoracotomy, preoperative FEV_1_ < 60%, preoperative hemoglobin score, duration of surgery ≥ 195 min, the amount of intraoperative blood loss per 100 mL, transfusion of packed red blood cells (PRBCs), transfusion of FFP, infusion of colloids, one-lung ventilation (OLV) duration ≥ 175 min, intraoperative crystalloids ≥ 6 mL/kg/h, and admission to the intensive care unit (ICU) with mechanical ventilation after surgery.

A *p*-value of ≤0.05 was deemed statistically significant. Statistical analysis was performed using IBM SPSS Statistics for Windows (Version 23.0, Armonk, NY, USA: IBM Corp.). We have also performed a post hoc power analysis based on the observed data, addressing the different in-hospital mortality rates between patients with PPCs and those without. To gain a power of 90% with an alpha error of 0.05, our post hoc power analysis revealed that each group required a minimum of 267 cases.

## 3. Results

**Figure 2:**
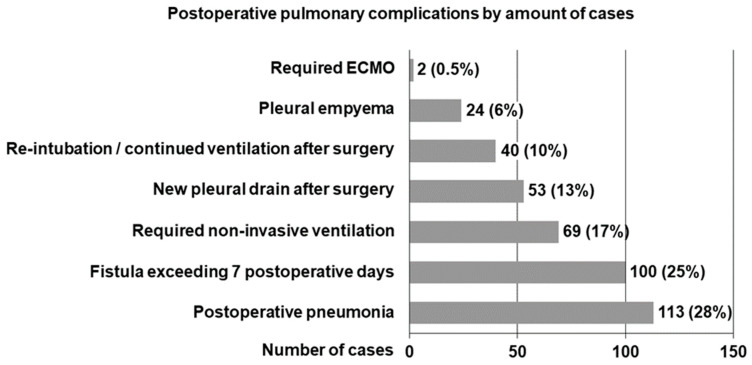
Rate of postoperative pulmonary complications according to case volume. ECMO = extracorporeal membrane oxygenation.

Data from 2.794 patients undergoing OTLR indicated that 750 cases (27%) had leastways one documented postoperative complication. Among the 1.368 patients who underwent OTLR for reasons other than primary lung cancer, 278 (20%) experienced one or more PPCs. In this subgroup, a total of 401 PPCs were identified, and their specific incidence is illustrated in Figure 2.

Table 1 presents the findings from the univariate analysis, which compares the preoperative characteristics of patients with specific conditions receiving OTLR for reasons other than primary lung cancer resection. The group with PPCs as well as that without PPCs are shown. Table 2 outlines the procedure-related risk factors, categorized by surgical and anesthesia-related traits, for patients undergoing OTLR for indications other than primary lung cancer resection.

As shown in Table 2, in-hospital mortality is significantly higher for patients who suffer from PPCs than for those who do not (7.2% vs. 1.5%; *p* = 0.000).

**Figure 3:**
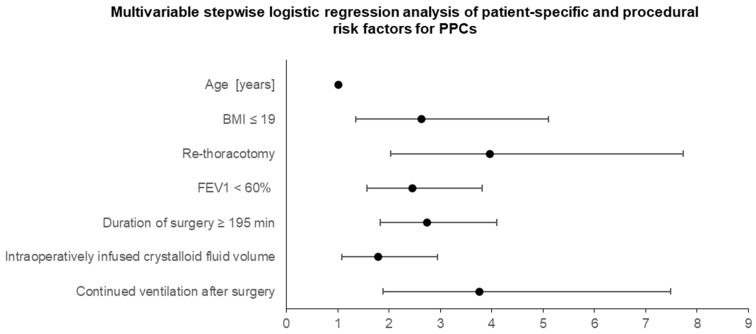
Multivariable stepwise logistic regression analysis of patient-specific and procedural risk factors for PPCs in patients undergoing thoracotomy for indications other than primary lung cancer. OR and 95% CI are shown. BMI= body mass index; FEV_1_ = forced expiratory volume in 1 s.

Figure 3 illustrates the results of the multivariable stepwise logistic regression analysis. The analysis identified significant patient-related risk factors such as increasing age over the years and BMI ≤ 19 kg/m^2^ (age: OR 1.02; 95% CI 1.00–1.03; *p* = 0.027; BMI ≤ 19: OR 2.6; 95% CI 1.4–5.1; *p* = 0.004).

Significant preoperative risk factors remained FEV_1_ < 60% and re-thoracotomy (FEV_1_ < 60%: OR 2.5; 95% CI 1.6–3.8; *p* < 0.001; re-thoracotomy: OR 4.0; 95% CI 2.0–7.8; *p* < 0.001).

The only surgical risk factor that remained significant was a duration of surgery ≥ 195 min (OR 2.7; 95% CI 1.8–4.1; *p* < 0.001).

In the multivariable logistic regression analysis, two anesthesia-related risk factors demonstrated a significant correlation with postoperative pulmonary complications (PPCs). First, admission to the intensive care unit (ICU) as an endotracheally intubated patient after surgery was shown to have an odds ratio of 3.8, (95% CI 1.9–7.5; *p* < 0.001). Additionally, the infusion of intraoperative crystalloids exceeding 6 mL/kg/h was associated with an OR of 1.8 (95% CI 1.1–3.0; *p* = 0.024).

## 4. Discussion

This multicenter registry analysis previously reported an overall incidence of PPCs following OTLR at 27%, without differentiating the indications for OTLR [8], within the previously reported range of 5% to 55% [9,14,15,16]. Also, patients who underwent OTLR for indications other than primary lung cancer experienced a significantly decreased rate of PPCs compared to those receiving OTLR for primary lung cancer. This could have its reasons in a shorter duration of surgeries as well as the smaller extent of lung resection in patients receiving OTLR for reasons other than primary lung cancer. While some authors report a low incidence of PPCs in patients undergoing OTLR for various diseases other than BC, the reported rates of PPCs in patients receiving OTLR for indications other than primary lung cancer in our study are still around 20% [18,19]. Different entities, such as metastasectomy for non-pulmonary cancer, non-oncologic indications, or different types of surgery, might account for the differences observed. Varying definitions of PPC or combined cardiopulmonary morbidity as an endpoint might render direct comparison difficult [18]. Nevertheless, our findings align with other studies analyzing similar cohorts [12].

In-hospital mortality is statistically significantly increased from 1.5% to 7.2% in patients with PPCs. It has often been shown that mortality rates rise with the extent of lung parenchyma that is resected [20,21]. The volume of lung tissue removal is a known risk factor for PPCs after OTLR [22,23]. In our study, patients with PPCs underwent wedge resection less often than those without (21% vs. 32%).

According to Miskovic et al., our study shows a significant increase in mortality rates in patients suffering from PPCs [24]. Our study’s overall mortality rate of 1.5% is in the range previously shown by Grapatsas et al. for patients receiving lung metastasectomy [19].

Significant patient-related risk factors likely leading to the development of PPCs in the multivariable regression analysis of our study were getting older year by year (OR 1.02 per year of getting older), BMI below 19 kg/m^2^, and FEV_1_ < 60%. This suggests a strong correlation between risk factors and complications, especially in high-risk patients and their deleterious effects. However, the univariate analysis showed that the patients in the PPC group were significantly younger (62 vs. 69). At first, we did not expect this finding, but it can also be seen that the patients in the PPC group have significantly more often a higher ASA status and are therefore more often severely ill already preoperatively. The multivariable regression analysis revealed that getting one year older is an independent risk factor for PPCs. This is in line with the existing literature [10,11,12].

Chronic pulmonary disease is one of the significant risk factors in elective lung resection surgery, and FEV_1_ < 60% could be identified as an independent risk factor for PPCs in this study [11]. The OR of 2.6 for BMI below 19 kg/m^2^ might have its origin in tumor cachexia. Tumor cachexia is common in patients suffering from colorectal carcinoma with 50–61% but is often overlooked, understudied, and uncured [25]. As the leading subgroup in our study were patients with metastasectomy, this finding could point to tumor cachexia and, therefore, less vulnerability. One possible consequence would be implementing preoperative nutritional screening and therapy of malnourished patients, as it is part of many prehabilitation programs.

Using multivariable logistic regression analysis, this study identified two surgery-related and two anesthesia-related independent risk factors for postoperative pulmonary complications (PPCs). The surgical risk factors included the need for a re-thoracotomy, which is almost non-modifiable, and a surgical duration exceeding 195 min. These factors highlight the importance of perioperative management strategies focused on minimizing surgical time and addressing the necessity for re-thoracotomy to reduce the risk of PPCs. Surgically complex and complicative surgeries, but also the potentially deleterious sequelae of prolonged one lung-ventilation often correlate with the duration and invasiveness of surgery, both potentially contributing to the occurrence of PPCs. In 2019, our working group found that more than 120 min of VATS surgery was an independent risk factor for PPCs [10]. Analogously, we have already shown that a duration of surgery exceeding 195 min in OTLR for lung carcinoma resection was an independent risk factor for PPCs [8]. The underlying factors are most likely the same.

Re-thoracotomy was among the leading risk factors for the development of PPCs. As this fact could not be shown in patients receiving OTLR for lung carcinoma resection, it seems that the present subgroup is more vulnerable to re-thoracotomy (Baar, 2023) as the postoperative care protocols do not differ between the patients receiving OTLR because of primary lung carcinoma and those who do not. Perhaps, re-thoracotomy is a significant risk factor in OTLR for reasons other than primary lung cancer as the amount of cases is around 3 times larger. Another possible explanation could be the surgery’s complexity, but further investigation is needed to prove that. In a study that only looked at patients with pleural empyema, Semmelmann et al. also showed that revision surgeries are an independent risk factor for PPCs [7]. In 2022, Petrella et al. showed similar findings in patients receiving elective oncologic thoracic surgery [26].

Similarly to our findings in patients undergoing OTLR for lung carcinoma resection, the anesthesia-related risk factors included the transferal of patients ventilated postoperatively to an intensive care unit and the infusion of intraoperative crystalloid fluids exceeding 6 mL/kg/h.

Postoperative residual curarization, hypothermia, and the need for bed rest to ensure a successful surgical outcome, along with upper airway diseases, are often named factors resulting in the need for postoperative ventilation. These factors do not generally apply specifically to thoracic surgery patients. Inferentially, when feasible and safe, on-table extubation should be considered for all patients after OTLR, provided that their neurological status and cardiorespiratory situation allow it.

Our study highlights that maintaining homeostasis focusing on the proper management of hemodynamics during surgery is important. We found that hypervolemia, indicated by crystalloid infusion rates exceeding 6 mL/kg/h, is associated with a higher incidence of PPCs in patients undergoing OTLR, regardless of whether the surgery is because of lung carcinoma or other indications [8]. Increased volumes of administered fluids are a key modifiable intraoperative risk factor for PPCs in lung surgery [13,27,28,29]. These studies were conducted in lung surgery patients who underwent either thoracotomy or VATS. Arslantas et al. identified that infusing more than 6 mL/kg/h intraoperatively is a risk factor for PPCs when analyzing patients experiencing thoracic surgery [13]. This threshold was also found to be a significant risk factor in our previous study, which looked at risk factors for PPCs after VATS lung cancer resections as well as after OTLR for lung carcinoma resection [8,14].

Various studies have recognized the ASA score as a significant predictor of PPCs following lung surgery, using an ASA score ≥ 3 as the cutoff [22,30]. Our study found that significantly more patients with PPCs had an ASA status of ≥3 in the univariate analysis, according to this threshold in the literature.

Our study has several limitations: The study has a retrospective character. The accuracy of the data presented relies on the available sources, which are different medical centers in Germany with different patient data management systems, and many individuals were involved in adding the data to the registry. Interpreting data from medical documents requires precise and comprehensive documentation. The study’s retrospective nature prevents establishing causal conclusions but allows for the identification of statistical associations that need further scientific validation. The multicenter design leads to differences in perioperative treatment; however, the large cohort aims to minimize these differences as well as reflecting standard medical practices. Based on the voluntary participation of thoracic surgery centers in this registry, it is, of course, the case that a particular selection bias is inherent in the study design. Only data from centers that have an intrinsic interest in evaluating and analyzing their treatment and outcome data were included in the present analysis. It is, therefore, important to place the available data in the context of the international literature. Of course, we must consider that different surgical techniques and surgeons’ different experience levels can have a relevant impact. Unfortunately, this is a design-inherent confounder of our multicenter findings and is not changeable.

Further prospective randomized studies are required to establish a causal relationship for the associations identified in our study. Additionally, the definitions of PPCs vary across different studies, even though the definition of postoperative pneumonia was taken from the EPCO guidelines. Other PPCs were not determined using a standardized classification system, nor were they based on the systematic classification of mortality and morbidity following thoracic surgery [17,31].

## 5. Conclusions

In spite of the abovementioned limitations, this study demonstrates the risk factors for PPCs in patients undergoing OTLR for indications other than lung carcinoma.

Primarily, a statistically significant increase in mortality can be seen when patients suffer from PPCs compared to that in patients without PPCs. A few independent risk factors for PPCs found in our study, for example, the patients’ age, cannot be changed by patients, surgeons, or anesthesiologists. Yet, performing extubation on the table (if promising) and crystalloid infusion not exceeding 6 mL/kg/h can be positively influenced completely or at least partially by the treatment team. They might help to reduce PPCs in patients undergoing OTLR for indications other than lung carcinoma. Preoperative decisions such as nutrition and training should also be taken into account. Due to the retrospective character of this study, future randomized trials need to focus on the suggestions created in our study.

## Figures and Tables

**Table 1:** Univariate analysis of patient-specific, preoperative characteristics that lead to risk factors for PPCs in patients undergoing open thoracotomy lung resection due to primary lung cancer. Data are presented as number of patients (percentage) or median (± interquartile range). PPCs = postoperative pulmonary complications; BMI = body mass index; CRP = C-reactive protein; ASA = American Society of Anesthesiology; FEV_1_ = forced expiratory volume in 1 s.

	No PPCs (*n* = 1.090)	PPCs (*n* = 278)	*p*-Value
Patient-Specific Characteristics			
Male gender	676 (62%)	186 (67%)	0.132
Age [years]	69 ± 15	62 ± 14	0.005
≥60 years of age	589 (54%)	166 (60%)	0.090
BMI [kg/m^2^]	27.2 ± 6.5	26.4 ± 6.1	0.079
BMI ≤ 19	46 (4%)	25 (9%)	0.001
BMI > 30	293 (27%)	61 (22%)	0.093
Smoking			
never	454 (42%)	117 (42%)	0.896
current	191 (18%)	57 (21%)	0.250
cessation ≥ 3 months	412 (38%)	78 (28%)	0.003
ASA ≥ 3	92 (8%)	56 (20%)	0.000
Preoperative Characteristics			
Chemotherapy	134 (12%)	38 (14%)	0.537
Radiation	72 (7%)	15 (5%)	0.461
Re-thoracotomy	218 (20%)	75 (27%)	0.011
Pulmonary infection < 4 weeks prior to surgery	76 (7%)	41 (15%)	<0.001
CRP < 3 mg/dL	158 (15%)	25 (9%)	0.016
Hemoglobin [mg/dL]	12.8 ± 2.3	11.9 ± 2.5	<0.001
pH	7.43 ± 0.04	7.43 ± 0.04	0.551
paO_2_ [mmHg]	77 ± 12	76 ± 14	0.237
paCO_2_ [mmHg]	37 ± 6	38 ± 6	0.333
FEV_1_ < 60%	123 (14%)	53 (28%)	<0.001

**Table 2:** Univariate analysis of surgery- and anesthesia-related characteristics that lead to risk factors for PPCs in patients undergoing open thoracotomy lung resection due to primary lung cancer. Data are presented as number of patients (percentage) or median (interquartile range). PPCs = postoperative pulmonary complications; PRBCs = packed red blood cells; OLV = one-lung ventilation; PEEP = positive end-expiratory pressure.

	No PPCs (*n* = 1.090)	PPCs (*n* = 278)	*p*-Value
Surgery-Related Characteristics			
Duration of surgery ≥ 195 min	168 (15%)	88 (32%)	<0.001
Pneumonectomy	7 (1%)	5 (2%)	0.065
Lobectomy	101 (9%)	35 (13%)	0.098
Bi-lobectomy	5 (1%)	2 (1%)	0.587
Segment resection	76 (7%)	24 (9%)	0.343
Wedge resection	351 (32%)	58 (21%)	<0.001
Intraoperative blood loss	315 ± 599	555 ± 671	<0.001
Partial ligation of pulmonary artery	22 (2%)	10 (4%)	0.120
Anesthesia-Related Characteristics			
Total intravenous anesthesia	520 (48%)	141 (50%)	0.370
Intraoperatively infused crystalloid fluid of crystalloids ≥ 6 mL/kg/h	834 (77%)	236 (85%)	0.003
Infused colloids	76 (7%)	35 (13%)	0.002
Intraoperative vasopressors	807 (74%)	230 (83%)	0.003
Recruitment maneuver	477 (44%)	109 (39%)	0.171
Transfusion of PRBCs	61 (6%)	51 (18%)	<0.001
Transfusion of fresh frozen plasma	20 (2%)	19 (7%)	<0.001
Continuous epidural or paravertebral analgesia	555 (51%)	157 (57%)	0.098
OLV PEEP ≤ 5 cmH_2_O	452 (47%)	101 (42%)	0.183
OLV PEEP ≤ 7 cmH_2_O	835 (86%)	199 (82%)	0.150
OLV duration ≥ 175 min	148 (14%)	69 (25%)	<0.001
Continued ventilation after surgery	66 (6%)	70 (25%)	<0.001
Mortality			
In-hospital mortality	16 (1.5%)	20 (7.2%)	0.000

## Data Availability

The original contributions presented in this study are included in the article/supplementary material. Further inquiries can be directed to the corresponding author.

## Supplementary Materials

The following supporting information can be downloaded at https://www.mdpi.com/article/10.3390/jcm14051565/s1, Table S1: STROBE statement.
